# A Case of Cancer of the Breast

**Published:** 1881-02

**Authors:** Wm. H. Fox

**Affiliations:** Oregon, Dane Co., Wis.


### Clinical Reports.

### Notes from Private Practice.

### Article VI.

*Case of Cancer of the Breast.—Recovery After Removal.*

In May, 1862, I amputated a cancerous breast for Mrs. Wm. Morehead, of Bellville, Dane county, Wisconsin. She was a very large and robust woman, aged forty-seven, the mother of one child, and appeared to be free from cancerous cachexia. Her breast was very large, and I cut widely of the disease, so as to extirpate any tissues that might possibly be implicated. Bleeding vessels were secured and the edges of the incision brought together by sutures, the whole being supported by a compress and bandage. The wound healed by the first intention, and the rapid recovery seemed complete.

In about two years after the operation, a scirrhus tumor about the size of a butternut appeared on the lower edge of the cicatrix and directly below the point where the center of the breast had been removed. I cut it out, brought the edges of the incision together by sutures, and applied a light compress and bandage. In a few days erysipelas appeared and spread rapidly, but was soon controlled, after which the wound healed quickly. From that time there was no recurrence of the cancerous disease, and Mrs. M. enjoyed good health. Her death, which occurred in May, 1880, was caused by umbilical hernia, eighteen years after the first and nearly sixteen years after the second operation.

The breast is preserved in alcohol. Some of the cellulo-adipose tissue, has become detached, and the breast appears to be much smaller than when first removed. Wm. H. Fox.

Oregon, Dane Co., Wis.

